# Supplementary figures and images for: Quantum Simulation of Molecules in Solution
Source: J Chem Theory Comput. 2022 Nov 9;18(12):7457–69. doi: 10.1021/acs.jctc.2c00974 (PMC9754316; doi:10.1021/acs.jctc.2c00974)

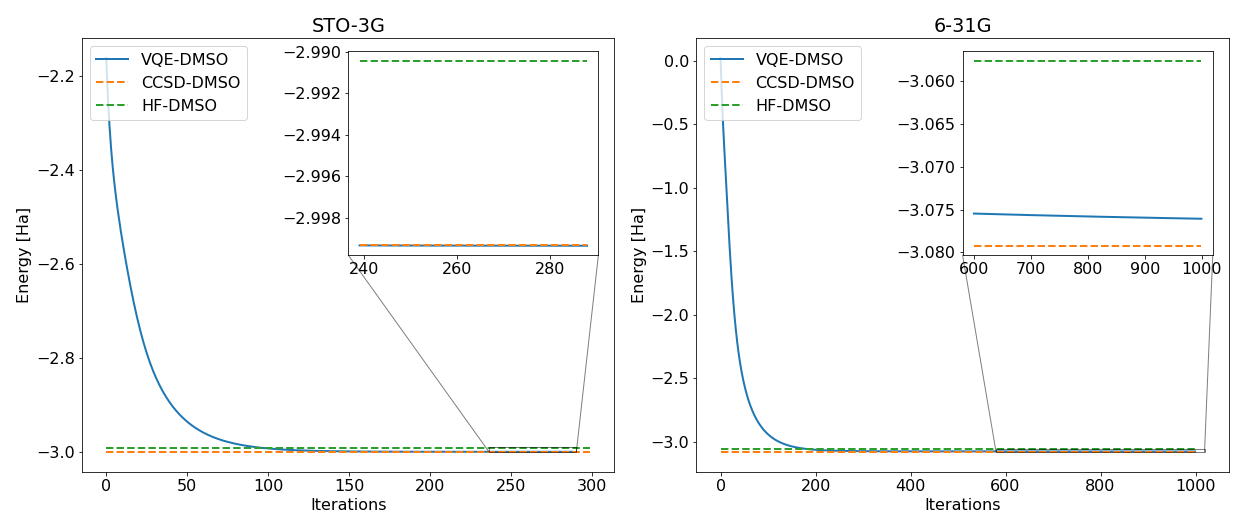

Supplement: Supplementary file 1 — ct2c00974_si_001.zip [file ct2c00974_si_001.zip › report_HeH+.png]

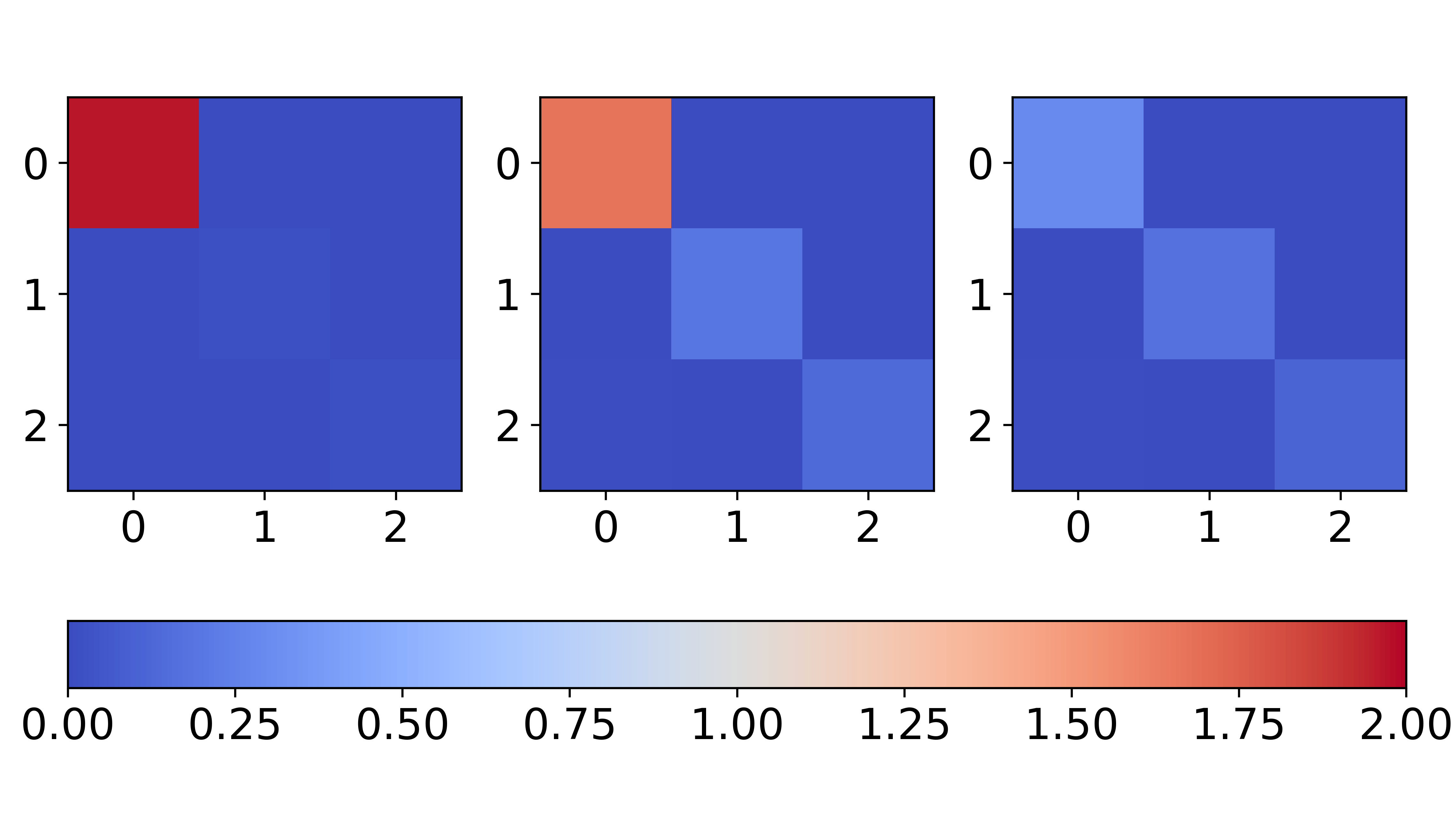

Supplement: Supplementary file 1 — ct2c00974_si_001.zip [file ct2c00974_si_001.zip › density_matrix_plot.png]

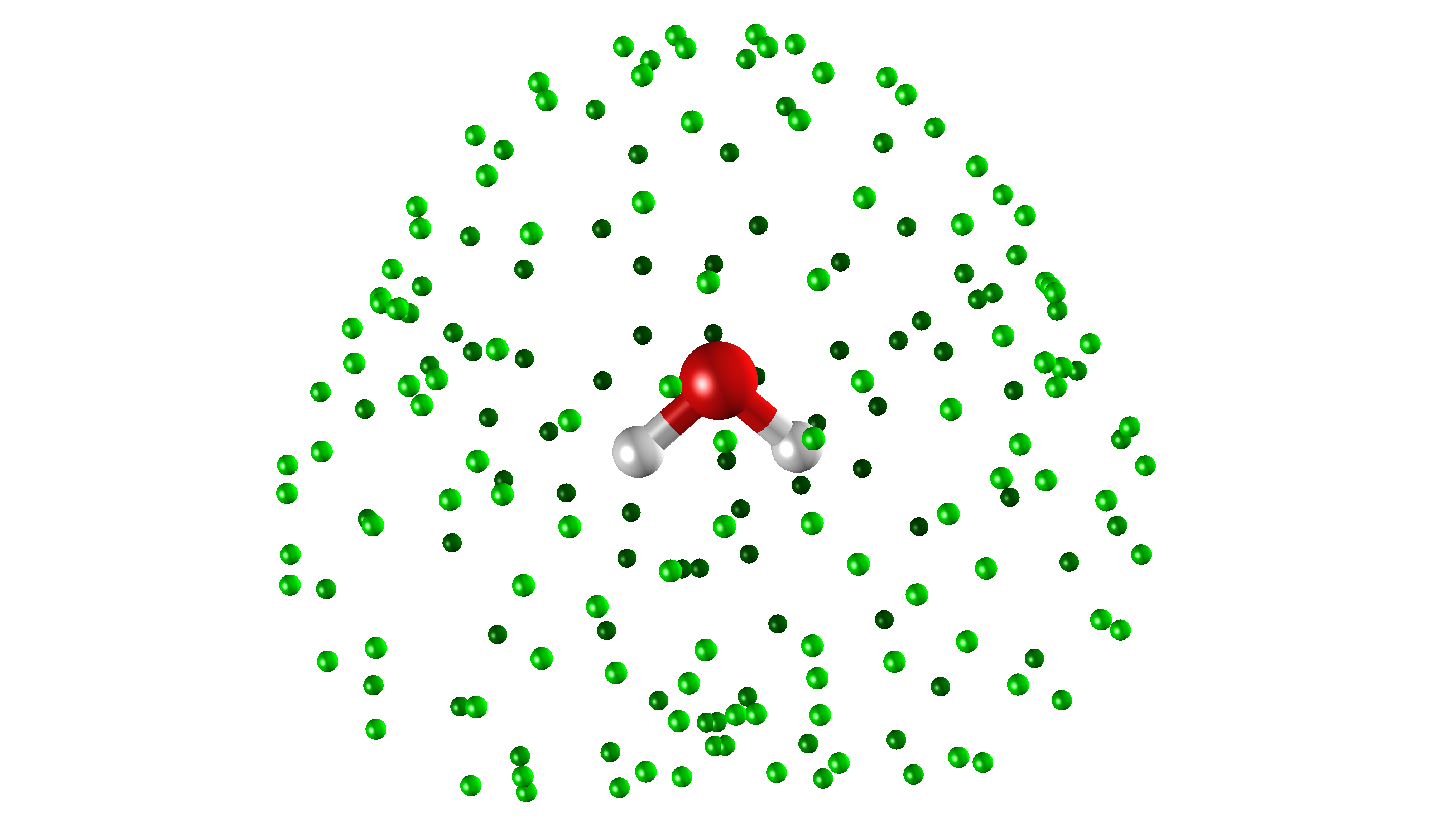

Supplement: Supplementary file 1 — ct2c00974_si_001.zip [file ct2c00974_si_001.zip › H20_cavity.png]

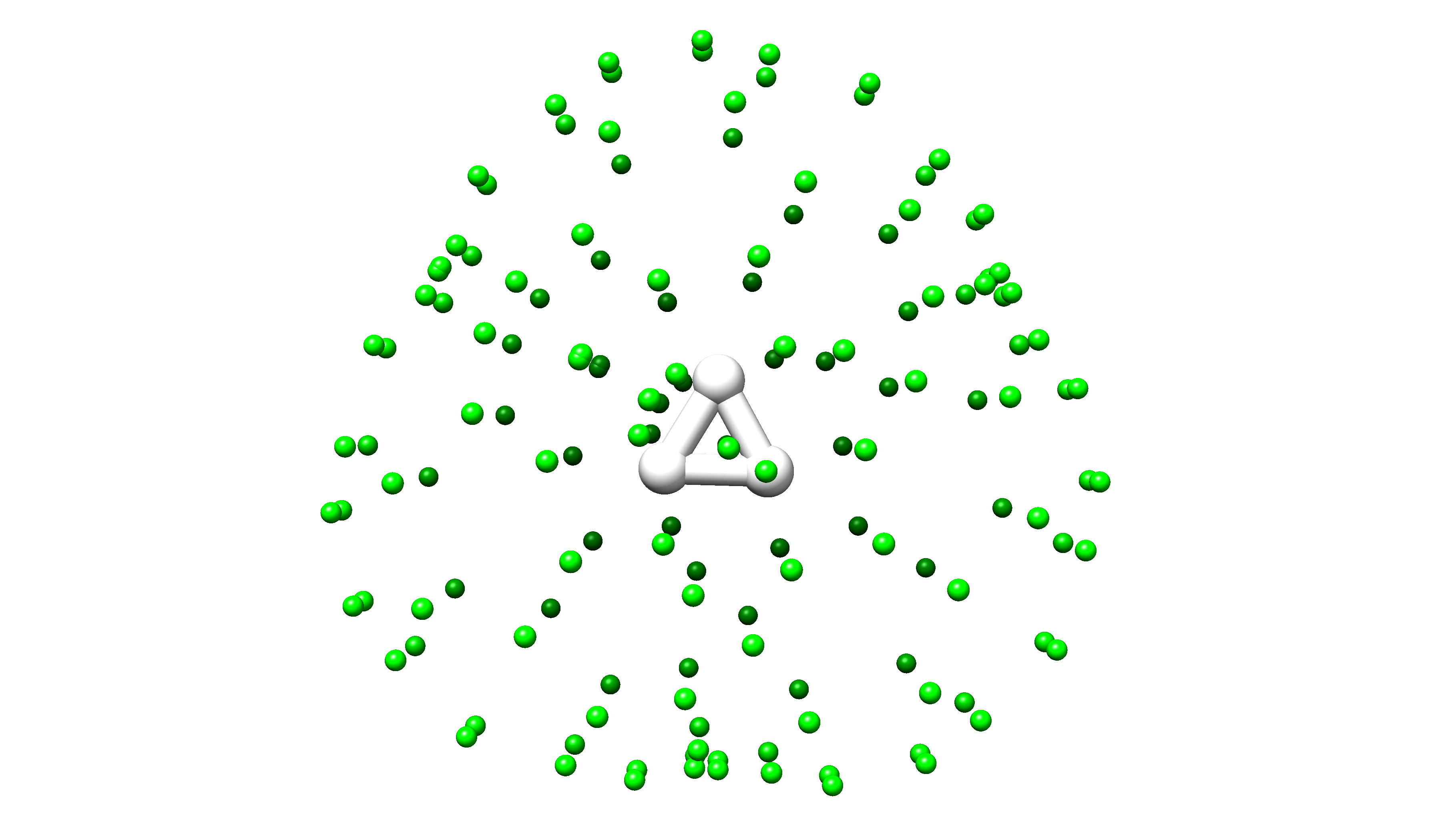

Supplement: Supplementary file 1 — ct2c00974_si_001.zip [file ct2c00974_si_001.zip › H3+_cavity.png]

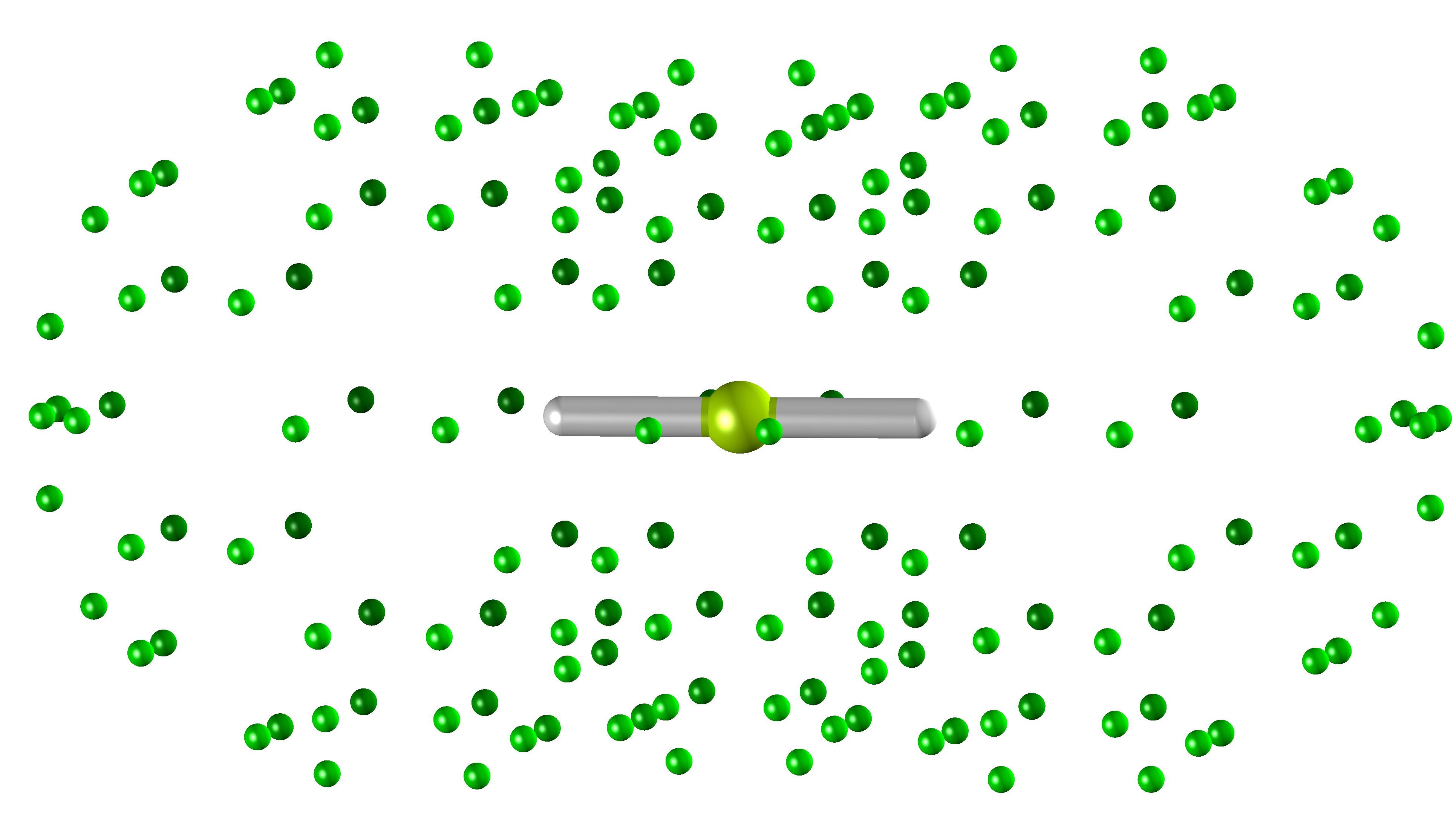

Supplement: Supplementary file 1 — ct2c00974_si_001.zip [file ct2c00974_si_001.zip › BeH2_cavity.png]

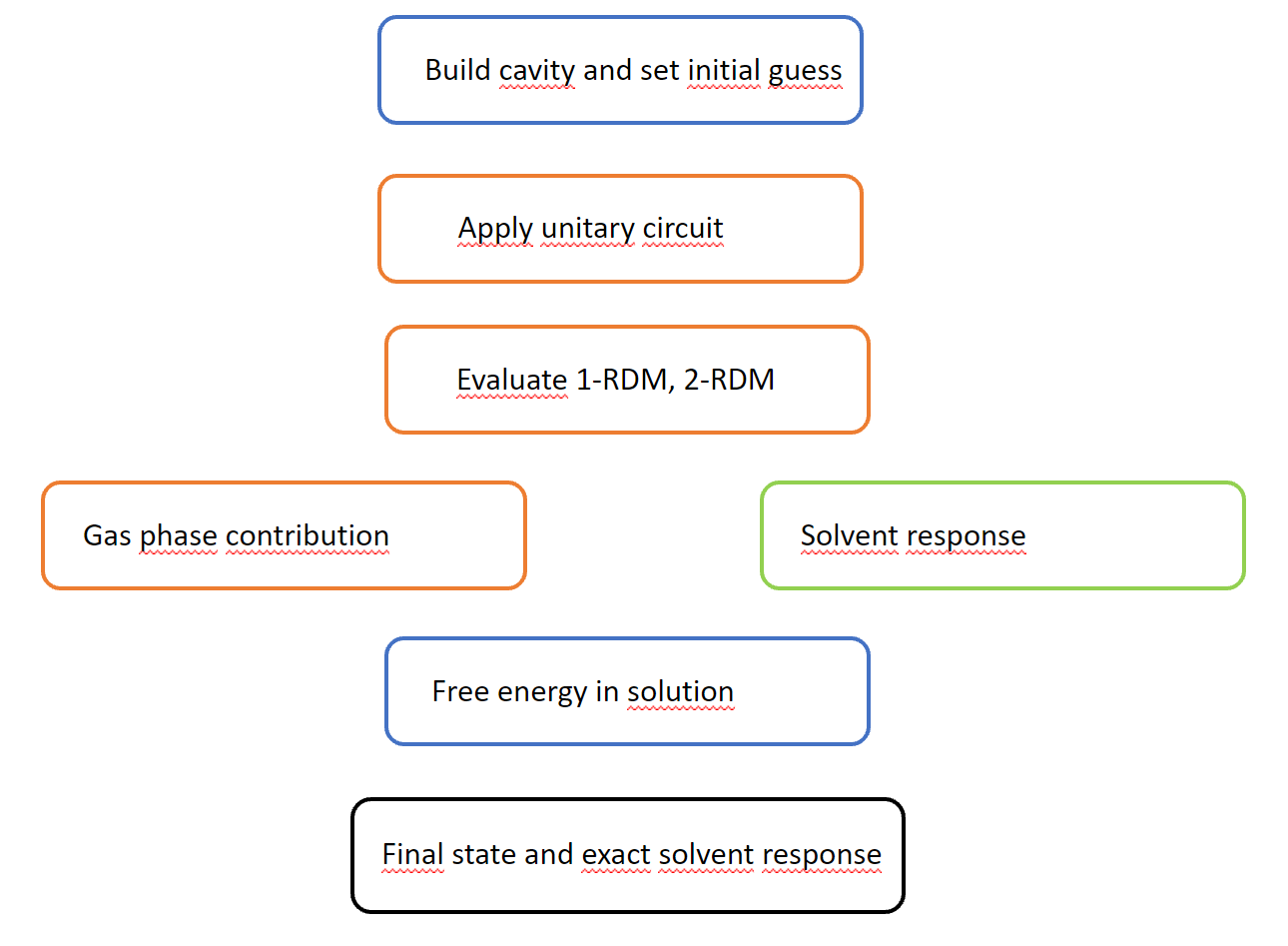

Supplement: Supplementary file 1 — ct2c00974_si_001.zip [file ct2c00974_si_001.zip › flowchart.PNG]

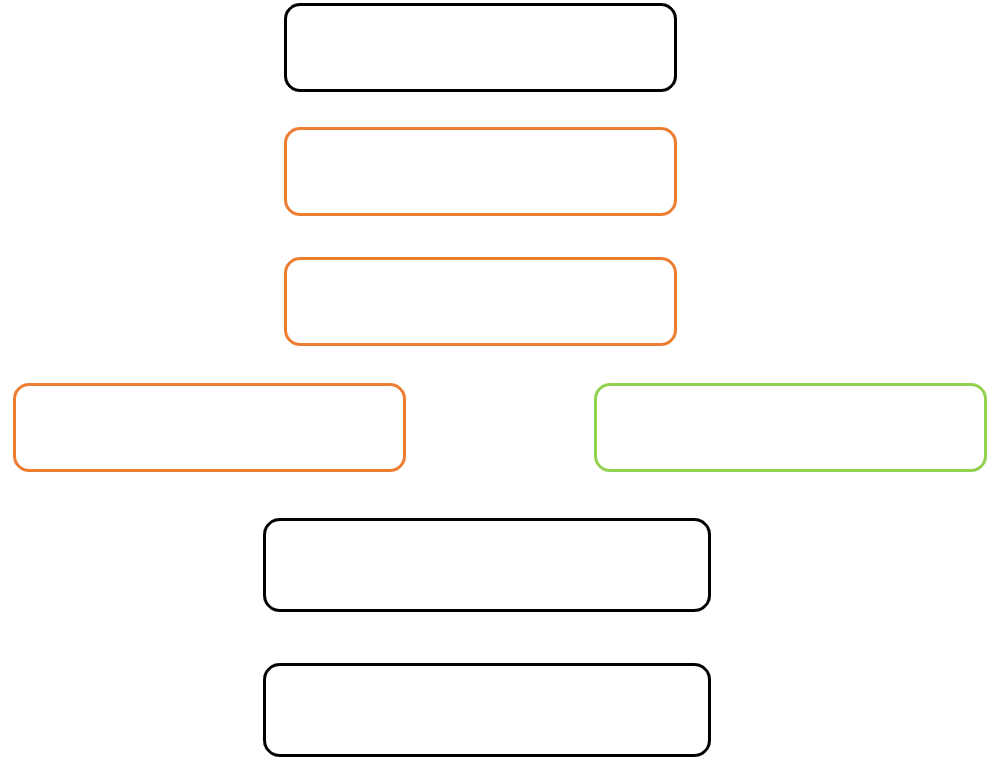

Supplement: Supplementary file 1 — ct2c00974_si_001.zip [file ct2c00974_si_001.zip › empty_flowchart.PNG]

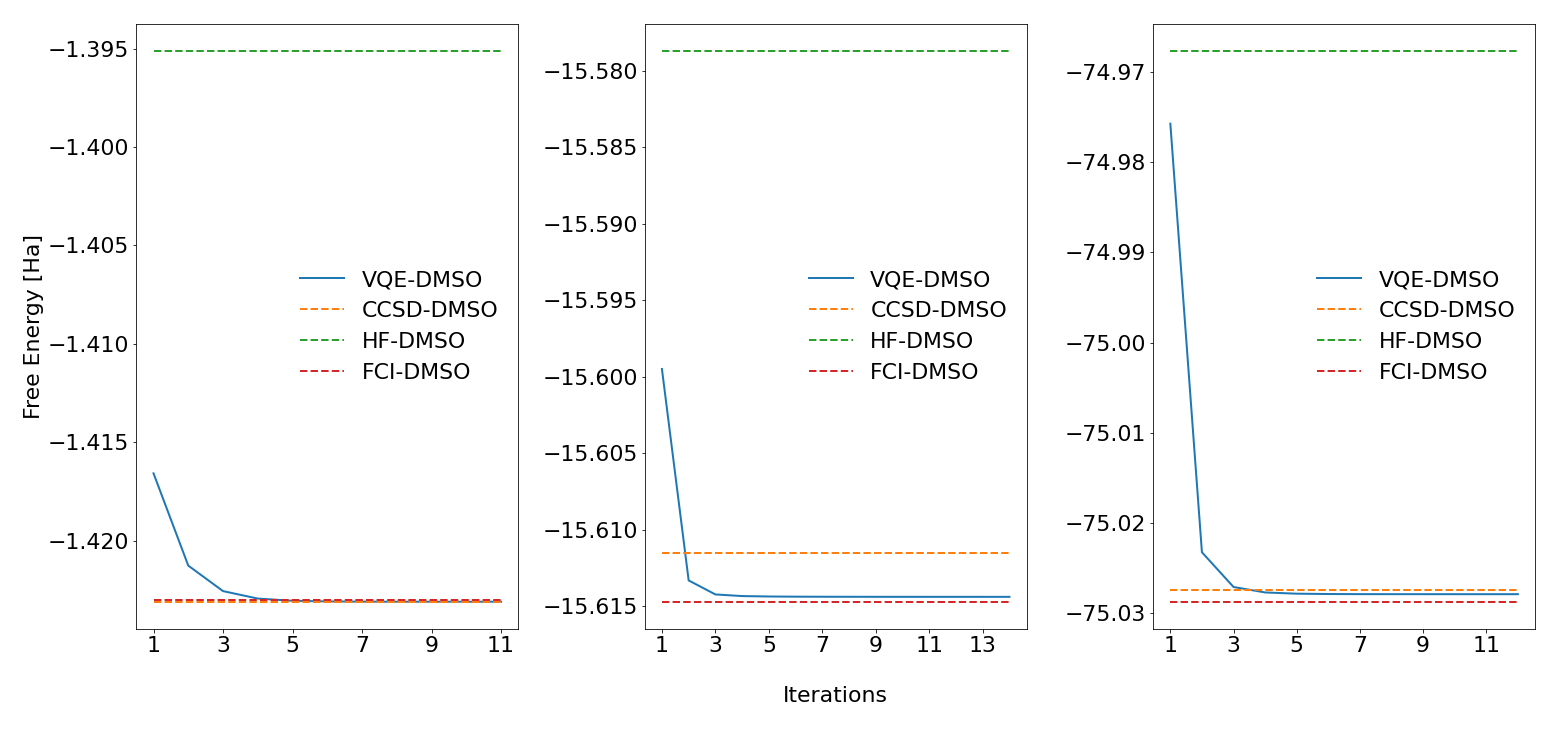

Supplement: Supplementary file 1 — ct2c00974_si_001.zip [file ct2c00974_si_001.zip › comparison_molecules_adapt_VQE_PCM.png]

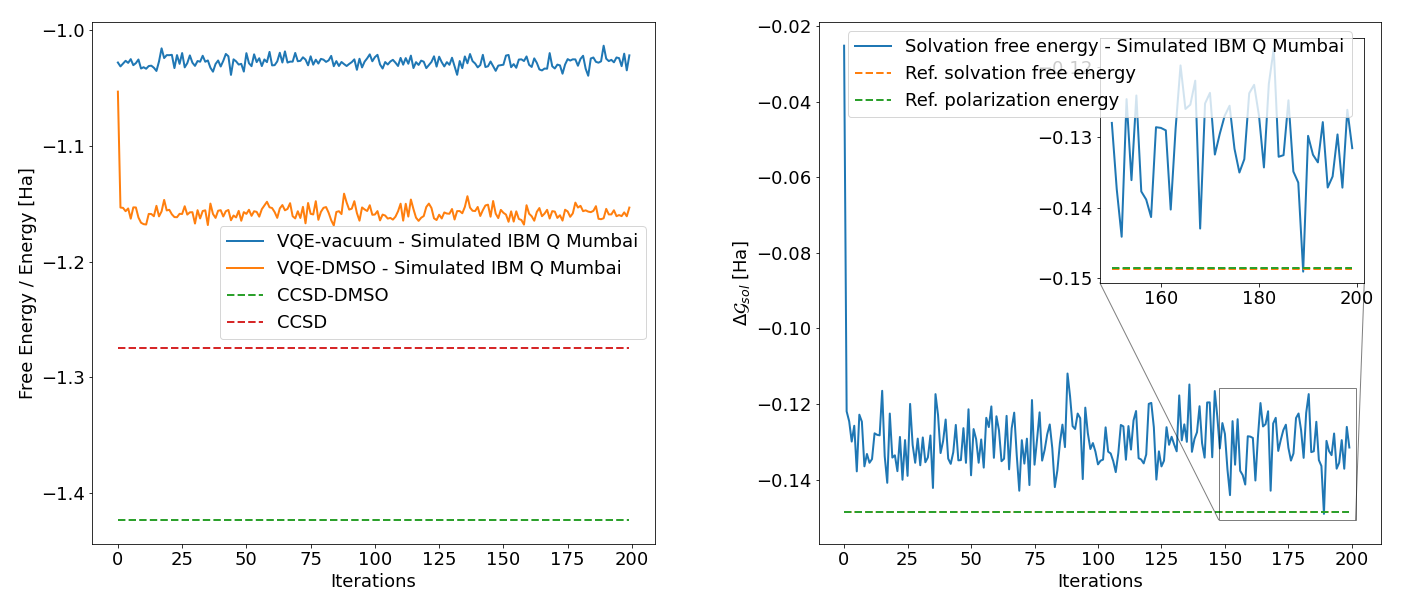

Supplement: Supplementary file 1 — ct2c00974_si_001.zip [file ct2c00974_si_001.zip › solvation_energy_plot_h3.png]

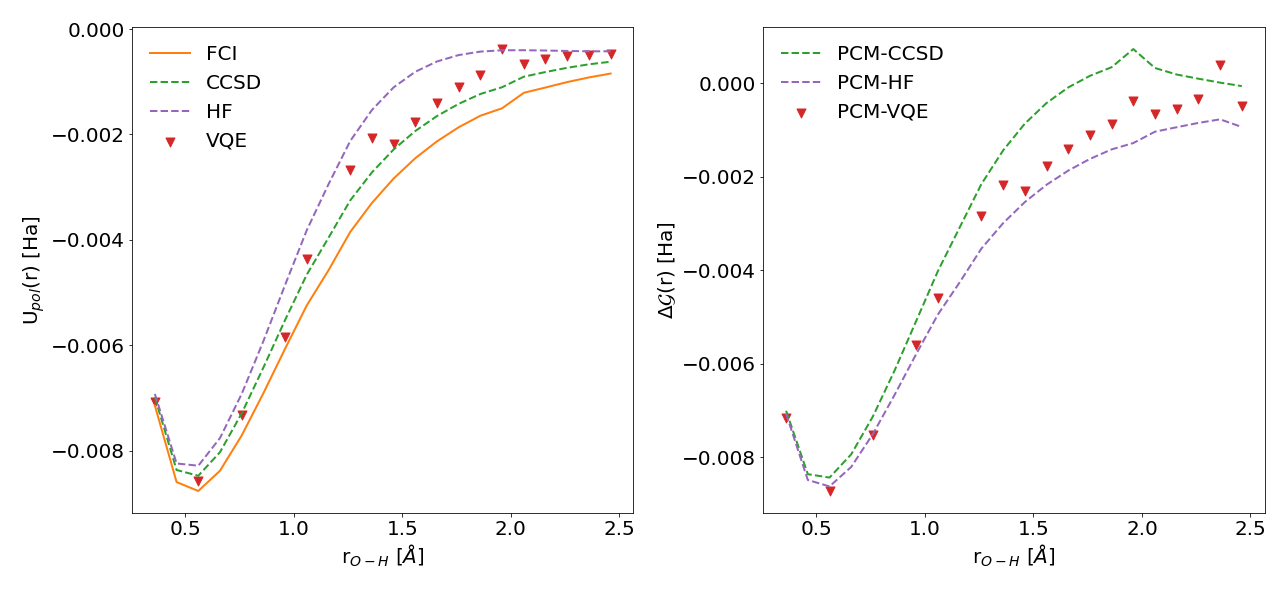

Supplement: Supplementary file 1 — ct2c00974_si_001.zip [file ct2c00974_si_001.zip › H2O_symmetric_stretching_solvation_properties.png]

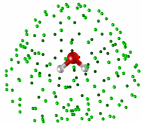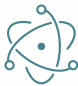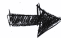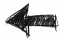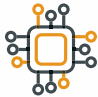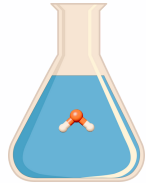

Supplement: Supplementary file 1 — ct2c00974_si_001.zip [file ct2c00974_si_001.zip › Graphical_abstract.pdf]

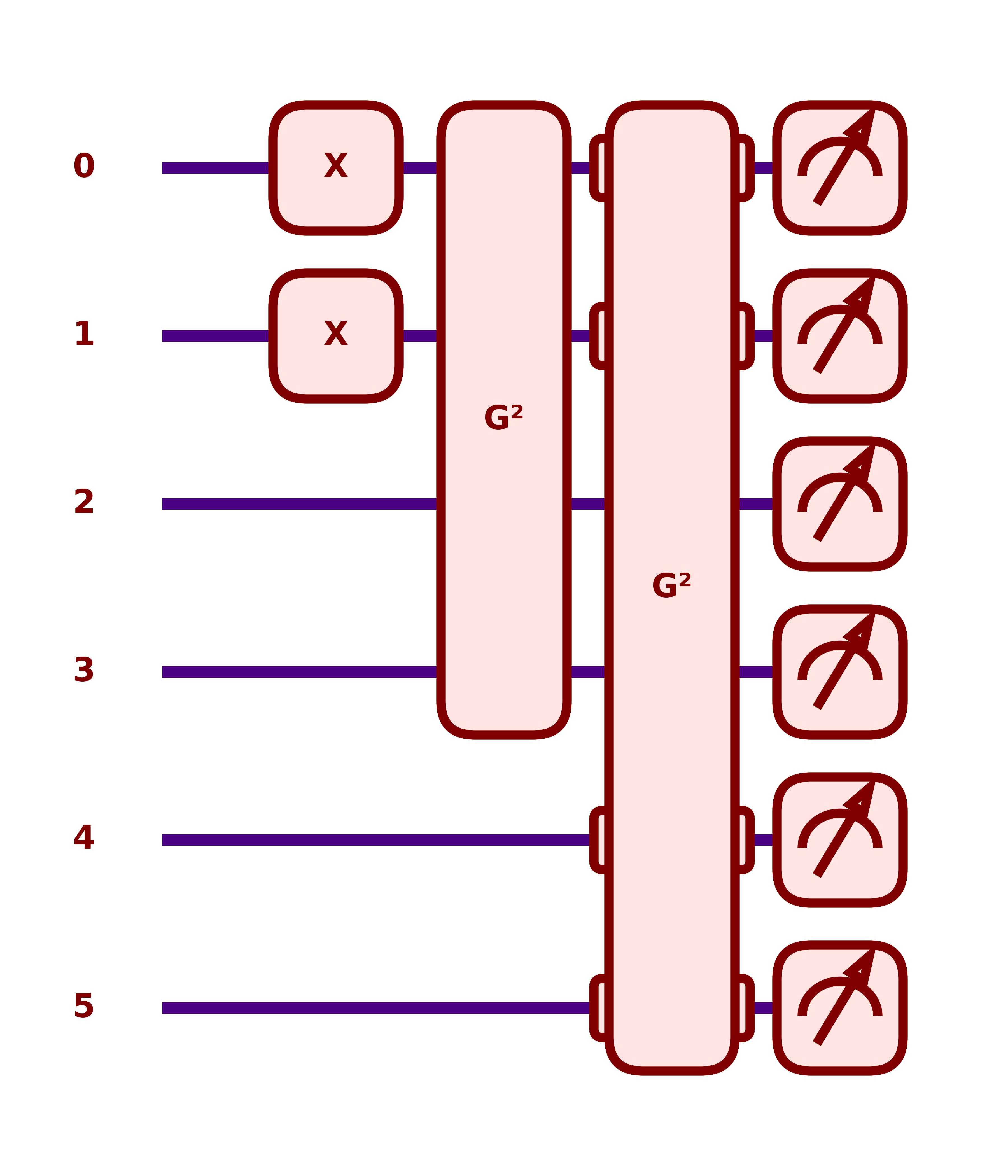

Supplement: Supplementary file 1 — ct2c00974_si_001.zip [file ct2c00974_si_001.zip › h3+_circuit.png]

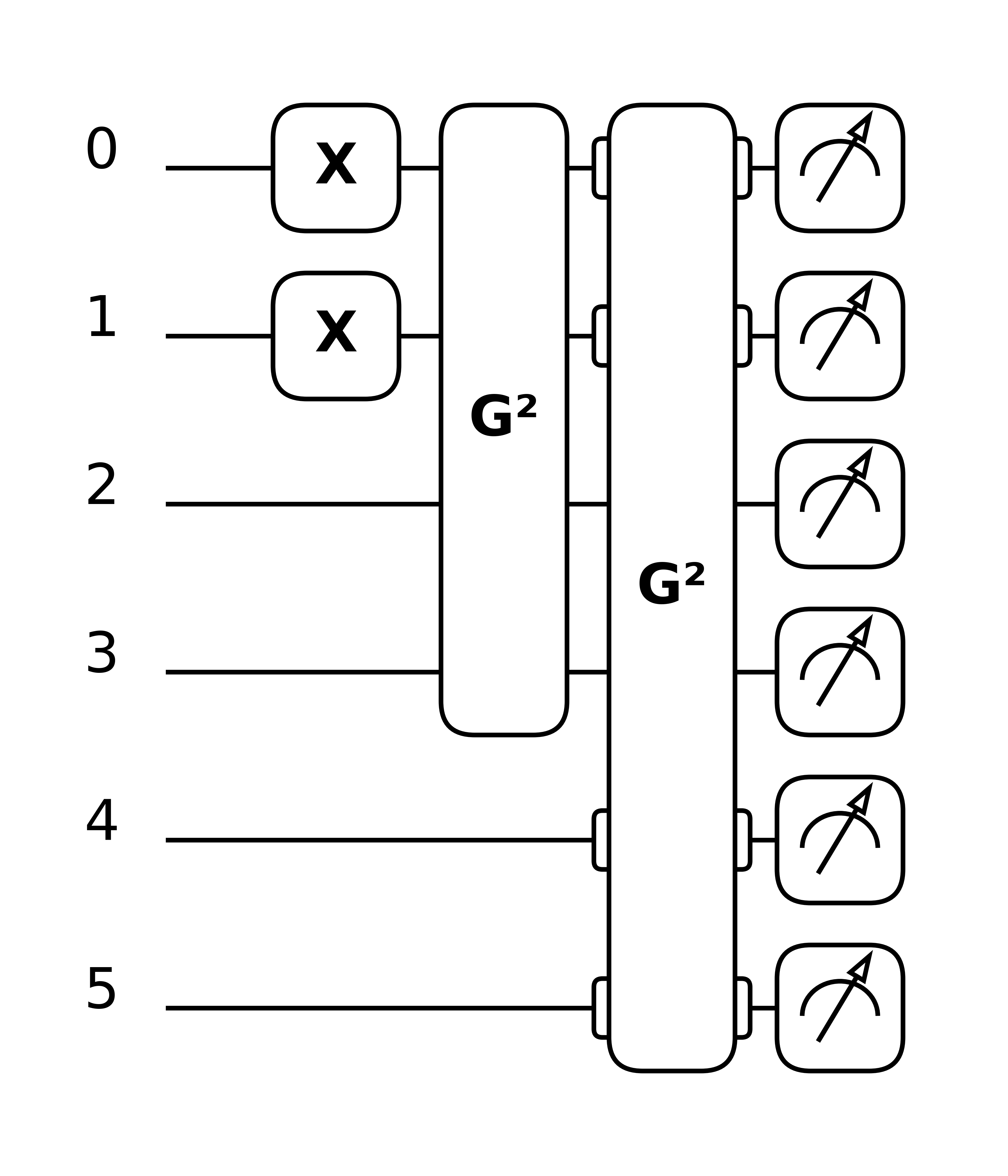

Supplement: Supplementary file 1 — ct2c00974_si_001.zip [file ct2c00974_si_001.zip › h3+_2_bw.png]

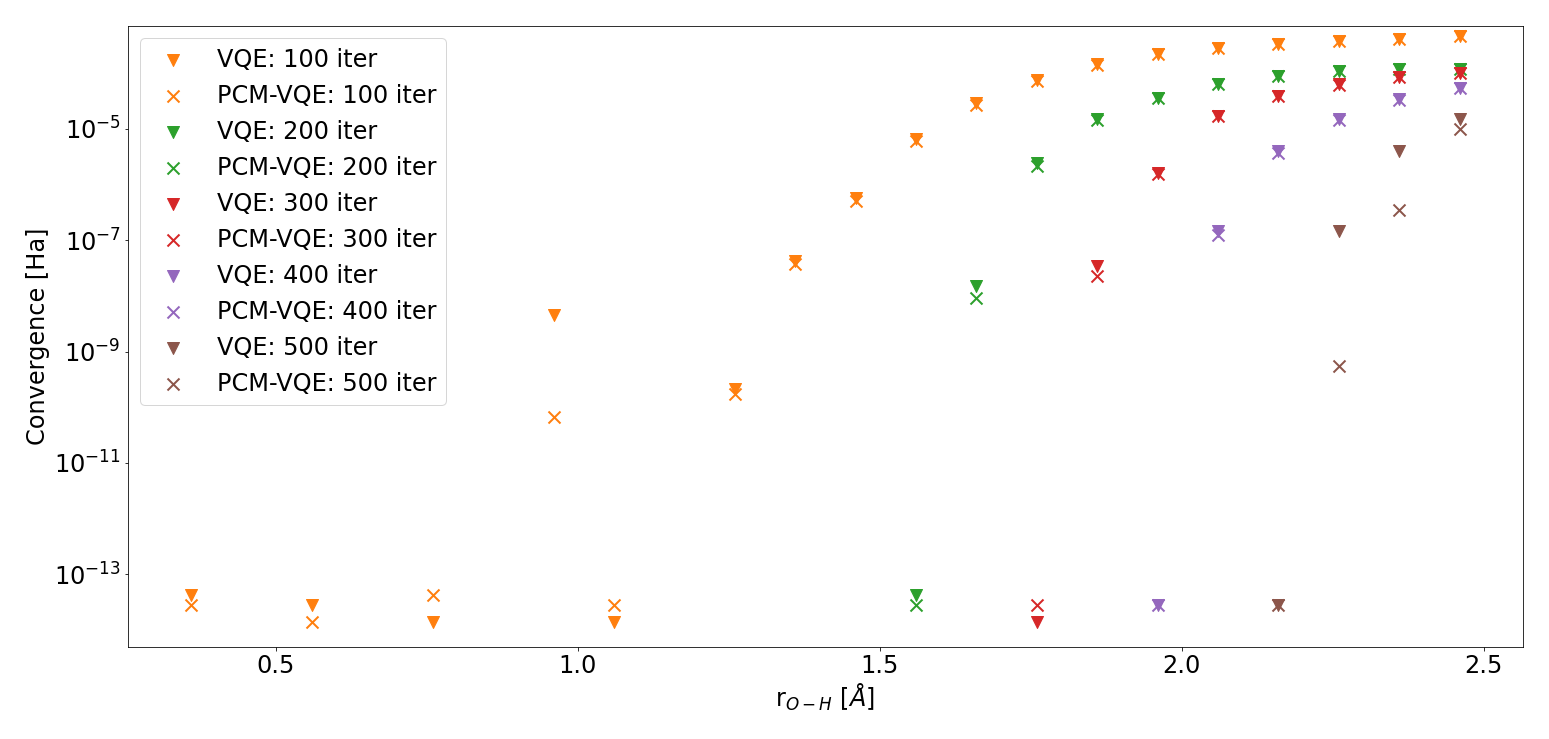

Supplement: Supplementary file 1 — ct2c00974_si_001.zip [file ct2c00974_si_001.zip › Convergence_plot_500_iterations_log_scale.png]

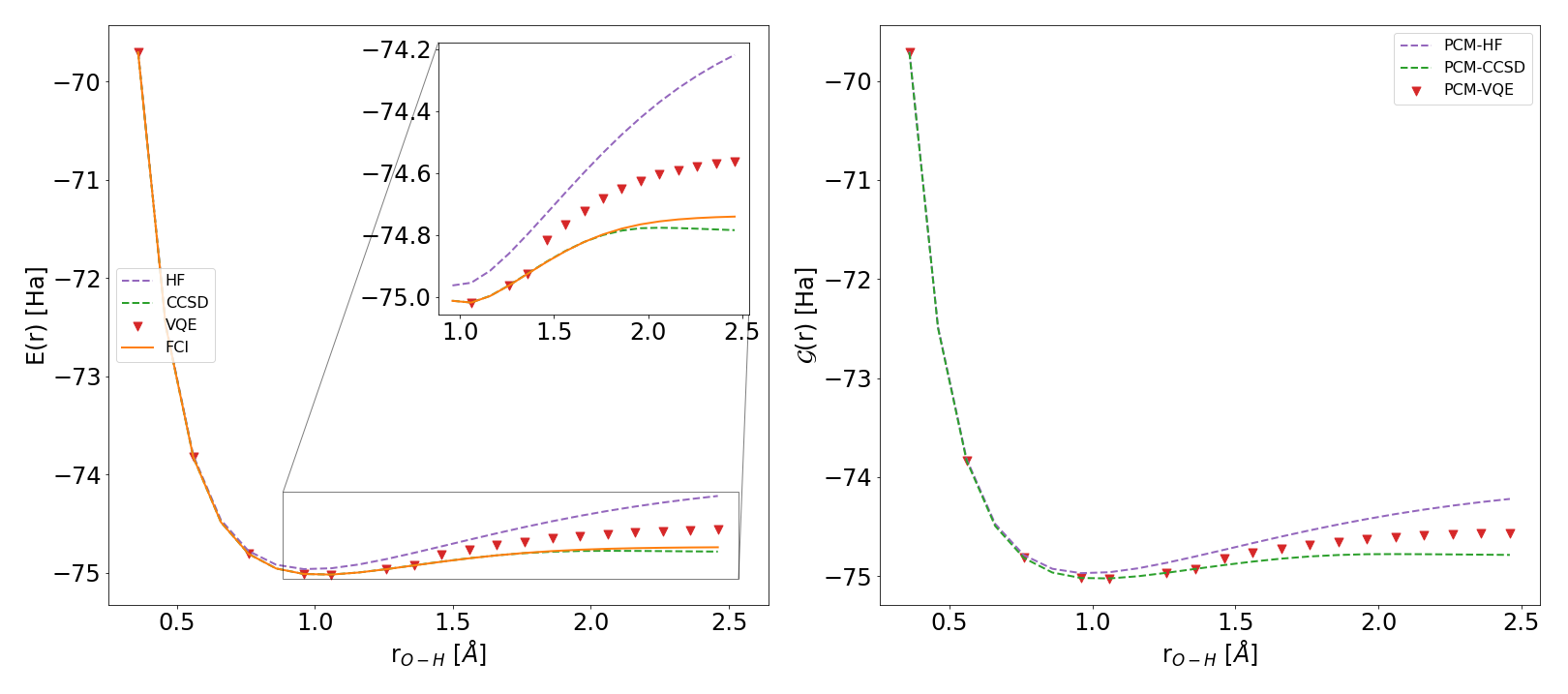

Supplement: Supplementary file 1 — ct2c00974_si_001.zip [file ct2c00974_si_001.zip › SI_water_double_dissociation_abs_energies.png]

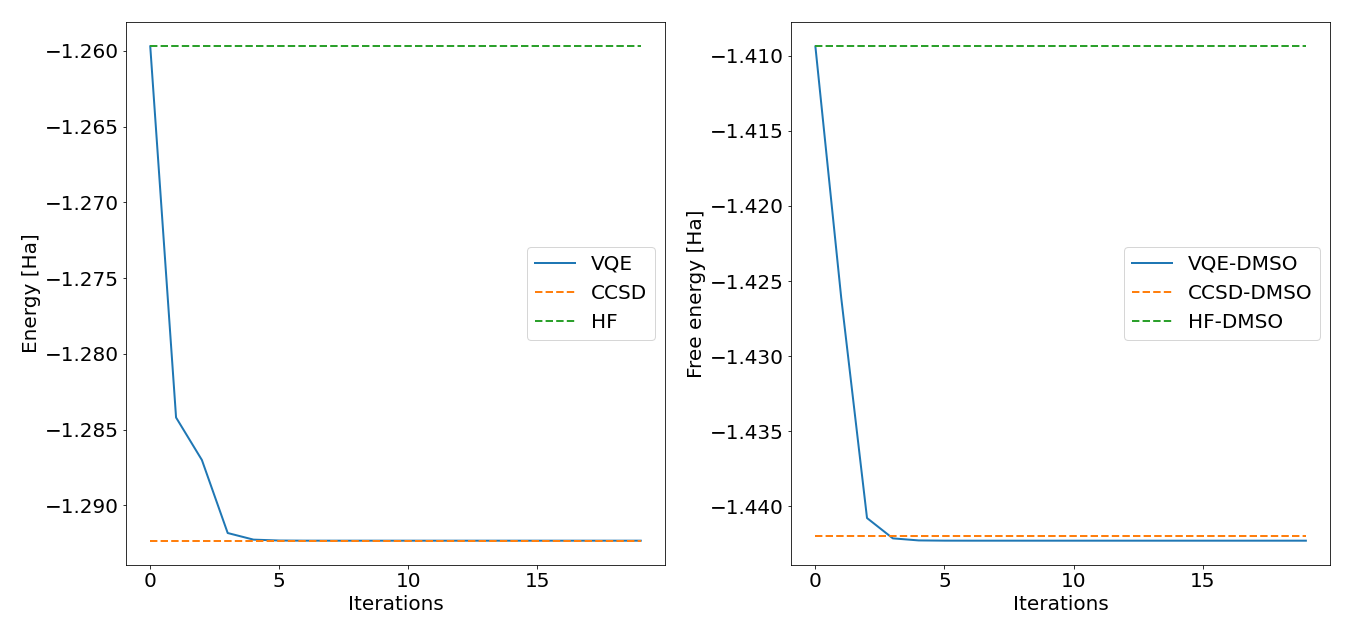

Supplement: Supplementary file 1 — ct2c00974_si_001.zip [file ct2c00974_si_001.zip › h3+_631_results.png]
