# Supplementary material for: Quantum Simulation of Molecules in Solution
Source: J Chem Theory Comput. 2022 Nov 9;18(12):7457–69. doi: 10.1021/acs.jctc.2c00974 (PMC9754316; doi:10.1021/acs.jctc.2c00974)
Supplement: Supplementary file 2 — ct2c00974_si_002.pdf [file ct2c00974_si_002.pdf]

Supporting Information for Quantum simulation of molecules in solution

Davide Castaldo,[†] Soran Jahangiri,[‡] Alain Delgado,[*,‡] and Stefano Corni[*,†,¶,§]

†*Università degli studi di Padova, Dipartimento di Scienze Chimiche, Via Marzolo 1 - 35131 Padova (Italy)*

‡*Xanadu, Toronto, ON M5G 2C8, Canada*

¶*Istituto Nanoscienze—CNR, via Campi 213/A, 41125 Modena (Italy)*

§*Padua Quantum Technologies Research Center, Università di Padova*

E-mail: alaindelgado@xanadu.ai; stefano.corni@unipd.it

Supporting Information Available

Pseudocode for the PCM-VQE implementation

Here we show a pseudocode scheme which summarizes the implementation of the PCM-VQE algorithm as it is implemented in our code.[1] Particularly this realizes a dynamical interface between Psi4/PCMSolver/PennyLane.

Our implementation allows to build the `Observable` and `Hamiltonian` objects of PennyLane with the data encoded in the Psi4 object `Wavefunction`. From the latter we have also access the PCMSolver routine that we leverage to update the polarization charges and to compute the corresponding value of polarization energy $\frac{1}{2}\langle\hat{V}_\sigma\rangle$. This last step summarized in the pseudocode as `Compute_sol_solv_int(d(`$\bar{\theta}_k$`), Q`$^{\mathtt{PCM}}$`)` is realized linking the call to the Psi4/PCMSolver function to the output of the `QNode` built with the PennyLane library.

Algorithm 1 PCM-VQE - Pseudocode of the implementation.

Require: Molecular_integrals, $\mathbf{Q}^{\text{PCM}}$ ▷ Psi4, PCMSolver
Ensure: $\mathcal{G}[\bar{\theta}]$, $\bar{\theta}$
  Initialization: ▷ PennyLane
  Fermion_to_qubit_mapping(Molecular_integrals)
  Set_Variational_Form()
  Set_Classical_Optimizer()
  k=0
  $\bar{\theta}_k \leftarrow \bar{\theta}_{guess}$
  while $k \leq max\_iter \;\; or \;\; \|\mathcal{G}[\bar{\theta}_k] - \mathcal{G}[\bar{\theta}_{k-1}]\| \leq \epsilon$ **do**
    Execute_variational_circuit($\bar{\theta}_k$) **return** $d(\bar{\theta}_k)$, $\langle \hat{H}_0 \rangle$ ▷ PennyLane
    Compute_sol_solv_int(d($\bar{\theta}_k$), $\mathbf{Q}^{\text{PCM}}$) **return** $\langle \hat{V}_\sigma(\bar{\theta}_k) \rangle_{\bar{\theta}_k}$ ▷ Psi4, PCMSolver
    $\mathcal{G}[\bar{\theta}_k] \leftarrow \langle \hat{H}_0 \rangle_{\bar{\theta}_k} + \frac{1}{2} \langle \hat{V}_\sigma(\bar{\theta}_k) \rangle_{\bar{\theta}_k}$
    k+=1
    $\bar{\theta}_k \leftarrow \bar{\theta}_{k+1}$ ▷ PennyLane
    $\mathcal{G}[\bar{\theta}_k] \leftarrow \mathcal{G}[\bar{\theta}_{k+1}]$
  end while
  $\bar{\theta} \leftarrow \bar{\theta}_k$
  $\mathcal{G}[\bar{\theta}] \leftarrow \mathcal{G}[\bar{\theta}_k]$
  return $\mathcal{G}[\bar{\theta}]$, $\bar{\theta}$

Finally PennyLane's optimizers update the variational parameters until the convergence criteria are met.

UCCSD Ansatz

Here we report additional results with a noiseless simulation concerning the calculation of the ground state energy of the HeH^+ molecule at the equilibrium distance in dimethyl sulfoxide (DMSO) (Fig. S1) using the PCM-VQE.

We have run these calculations to test the hybrid algorithm also on a well established ansatz such as the Unitary Coupled Cluster with Singles and Doubles (UCCSD). We recall that in this approach the optimization parameters coincide with the cluster amplitudes of the single and double excitation operators:

$$\hat{U}(\bar{\theta}) = e^{\hat{T}_1(\bar{\theta}) + \hat{T}_2(\bar{\theta})} \quad , \quad \begin{cases} \hat{T}_2(\bar{\theta}) = \sum_{i>j>k>l} \theta_{ijkl} (a_k^\dagger a_l^\dagger a_j a_i - a_j^\dagger a_i^\dagger a_k a_l) \\ \hat{T}_1(\bar{\theta}) = \sum_{i>j} \theta_{ij} (a_j^\dagger a_i - a_i^\dagger a_j) \end{cases} \tag{S1}$$

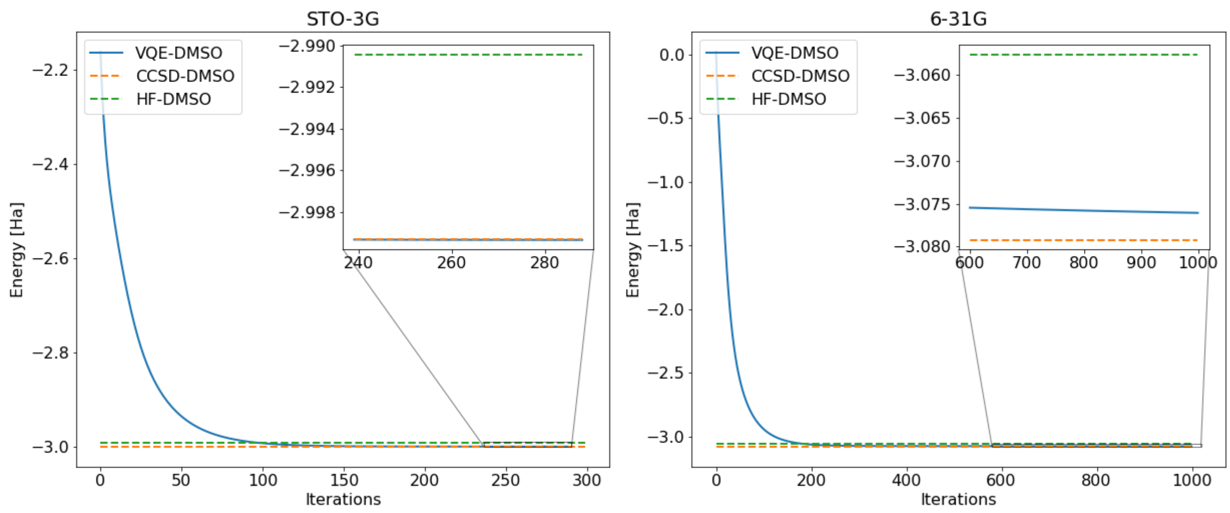

Figure S1: Ground state energies of the HeH^+ molecule in DMSO. (Left panel) The solid blue line represents the energy obtained with the PCM-VQE as a function of the iterations using a STO-3G basis set. As a reference we show the energy obtained with an HF-PCM calculation (green dashed line) and with a CCSD-PCM calculation (orange dashed line) using the same basis set. (Right panel) The same plot is reported adopting the 6-31G basis set.

The implementation of the previous unitary is done performing a Trotterization since the two excitation operators do not commute. The order of the Trotterization determines the number of control parameters and the depth of the circuit, here we employed a first order approximation.[2] The circuit template is provided by default in the PennyLane library.

Moreover, here we wanted to explore not only a different ansatz but also the effect of a larger basis set. For this reason we chose to investigate HeH^+, as the smallest system exhibiting substantial impact by the presence of a solvating environment, with the STO-3G basis and with the 6-31G basis set. As for the results shown in the main text, the initial state is the Hartree-Fock wavefunction, the molecular wavefunction is mapped onto the quantum computer state with the Jordan-Wigner method and the variational network parameters have been optimized with the same adaptive gradient descent algorithm. The variational parameters are initialized at random.

With regard to the quantum resources required for this calculation the helium hydride wavefunction with a STO-3G basis is mapped in a four qubits' quantum register, on the

other hand for the calculation in the 6-31G basis eight qubits are needed.

As we can see we are able to obtain good results recovering for the STO-3G basis set calculation (left panel) $> 99\%$ of the free energy in solution with respect to the exact calculation, while, for the 6-31G basis set (right panel), we reach the 85.4%. It is important to notice that, in this case, the classical optimization algorithm did not meet the convergence criteria and the result could be further improved by increasing the number of iterations.

These results further contribute to show that the performance of the PCM-VQE is similar to what has already been reported in literature for the UCCSD *in vacuo*[3,4] and that the inclusion of solvation effects does not impact the overall algorithmic efficiency.

H_3^+ calculations with PCM-VQE/6-31G

Here we report additional results on the H_3^+ molecule using the 6-31G basis set in DMSO (see Fig. S2). To perform the calculations we have adopted an adaptive ansatz including only the relevant excitations that contribute to the ground state wavefunction as described in the computational details' section of the main text. We have also reported the circuit structure in Fig. S6. As we can see in Fig. S2 the adaptive ansatz is able to reproduce exactly the energy in vacuum. Indeed we recall that for a two electron system the CCSD calculation is equivalent to Full Configuration Interaction.

Moving to the results for the solvated molecule (right panel) again we can see a very good agreement between the PCM-CCSD and the PCM-VQE calculations but in contrast with the gas phase result the numerical values are not exactly matching. Particularly, the PCM-VQE free energy is 8 meV lower. This is due to the fact that for the solvated system the PCM-CCSD calculation is performed approximating the reaction field at the HF level (PTE approximation in the original reference[5]) as implemented in Psi4.[6] Interestingly this effect is not noticeable in the STO-3G results of the main text because adopting a smaller basis set the error due to the PTE approximation is negligible.

Finally, it is worth to comment the convergence rate of this calculations w.r.t. the results obtained with a 6-31G basis set in the previous subsection (Fig. S1). As expected, the number of iterations and the convergence rate of the optimization procedure is dramatically improved using the adaptive ansatz instead of a less chemically-aware UCCSD circuit. This is in agreement with what already discussed in literature: the expressibility[7] of a parameterized quantum circuit affects the convergence properties of the classical optimizer due to wider Hilbert space sectors explored[8] w.r.t. adaptive circuits and to the emergence of barren plateaus.[9]

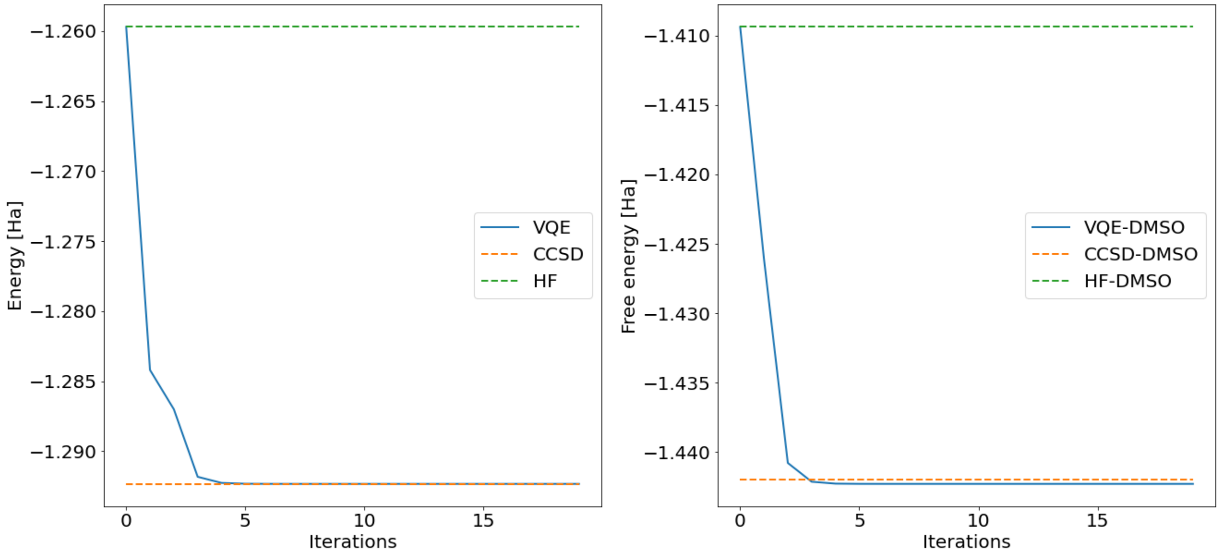

Figure S2: Ground state energies of the H_3^+ molecule with a 6-31G basis set. (Left panel) VQE results *in vacuo*; (Right panel) PCM-VQE results in DMSO. Solid blue line represents the energy obtained from the trial wavefunction as a function of the iterations. As a reference we show the energy obtained with an HF-PCM calculation (green dashed line) and with a CCSD-PCM calculation (orange dashed line) using the same basis set.

Additional results in aqueous solution

In this section we show additional calculations performed to test the PCM-VQE on the same systems studied in the main text but with a different solvent, i.e. water. These results, in accordance with what already discussed for the case of a DMSO solution, allow us to discuss

the performance of the algorithm on another very common solvent in the chemical practice.

Single point calculations

In Tab. S1 we report the results obtained running a PCM-VQE calculation in water. Analogously to what discussed in the main text for the case of DMSO, with the PCM-VQE we are able to reproduce the exact results reported for the CCSD-PCM calculation of the H_3^+ molecule and to recover a value of free energy in solution for the larger molecules (BeH_2 and H_2O) close to the total FCI energy comprehensive of the polarization energy contribution. All the calculations have been carried out according to the computational protocol described in the computational details' section referring to the results shown in the main text.

Table S1: Solvation free energies in water solution for the studied systems. Comparison between IEF-FCI/IEF-CCSD/PCM-VQE (H_2O). Molecular geometries for the calculations have been taken from Ref.[10] Energy values are reported in eV.

	FCI-PCM^a	CCSD-PCM	PCM-VQE
$\Delta\mathcal{G}_{H_3^+}$	-4.082	-4.082	-4.082
$\Delta\mathcal{G}_{BeH_2}$	-0.539	-0.468	-0.545
$\Delta\mathcal{G}_{H_2O}$	-0.142	-0.117	-0.133

(a) For FCI-PCM, polarization energies are reported rather than solvation free energy.

Energy and free energy plot for the H_2O double dissociation profile

Here we report additional results regarding our analysis on the polarization energy and hydration free energy discussed in the main text for the double bond dissociation profile of the water molecule. In Fig. S3a-b we report, respectively, the total energy (Fig. S3a) and the free energy in solution (Fig. S3b) computed with the PCM-VQE (red triangles). The energies reported correspond to an optimization convergence rate $\epsilon < 10e^{-8}$ or to a maximum number of 500 iterations. In this case, while maintaining the same trend as observed for U_{pol} and $\Delta\mathcal{G}$, we can see that the differences are considerably more pronounced for the total energy E and the free energy in solution $\mathcal{G}$. In particular, while up to 1.4 Å the (PCM-)VQE

result is quantitatively in agreement with that of the FCI (orange solid line) or CCSD (green dashed line), for higher bond length values we observe deviations from the value calculated by the FCI/CCSD method of about 0.2 Ha.

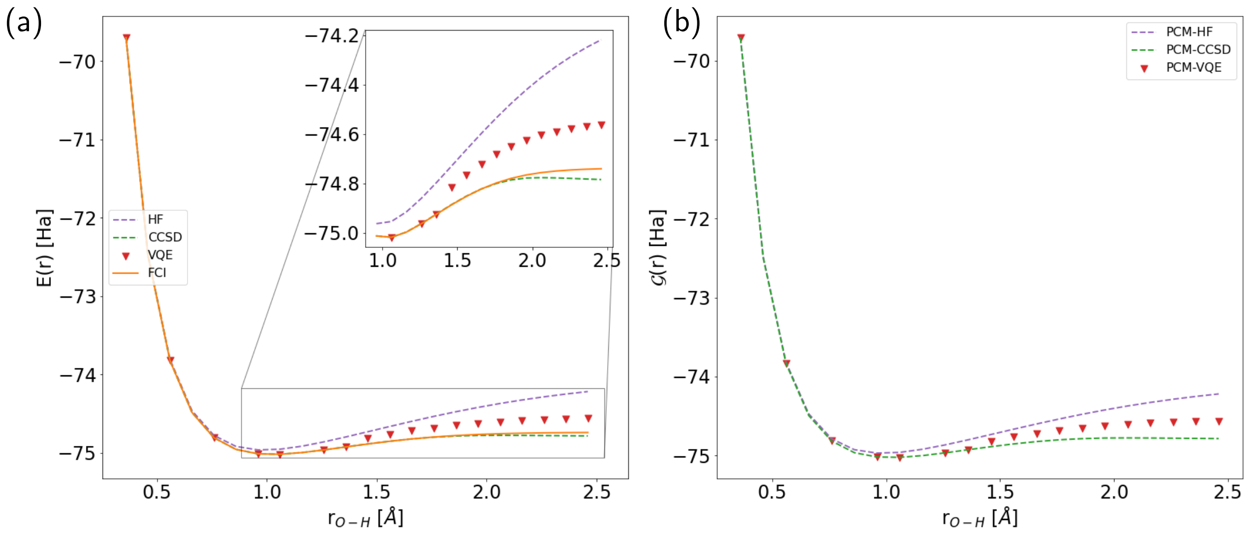

Figure S3: Double bond dissociation profile for the water molecule in gas phase (a) and in water (b). Reference values with classical methods are reported: FCI (solid orange line), CCSD (green dashed line) and HF (purple dashed line). Red triangles show the results obtained with the (PCM-)VQE method. All the calculations were performed with a STO-3G basis set.

Finally, we comment the data reported in Fig. S4 which show the convergence behavior of the classical optimization procedure as a function of the iterations (different colors) and as function of the bond length r_{OH}. As a first comment we can notice that the converge rate of the optimization procedure for the PCM-VQE (crosses) is very similar to the one observed *in vacuo* (triangles) in accordance with the plots shown in the main text for the single point calculations and with the results shown in Fig. S1 suggesting that the inclusion of a non-linear term in the cost functional should not further affect the classical optimisation algorithm.

Finally, we also notice that the rate of convergence of the optimization slows down considerably as the bond distance increases. This behavior is not surprising and can be explained considering (i) that as the dissociation regime approaches the initial guess state

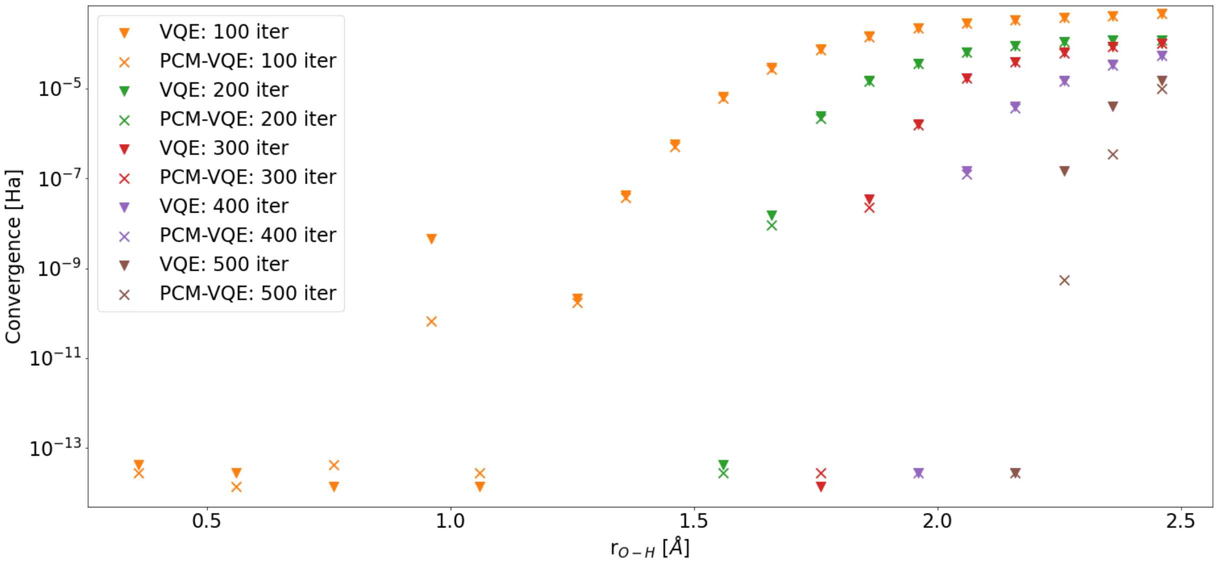

Figure S4: Convergence plot of the classical optimization procedure for the VQE (triangles) and PCM-VQE (crosses) as a function of the bond length. Color code: yellow ($\leq$ 100 iter.); green ($\leq$ 200 iter); red ($\leq$ 300 iter.); brown ($\leq$ 400 iter.); purple ($\leq$ 500 iter.).

$|HF\rangle$ provides a worse approximation of the optimal result and therefore a greater number of iterations is required to achieve convergence. (ii) The adaptive ansatz here used includes excited configurations among the most relevant to capture electronic correlation at the equilibrium geometry whose electronic structure is increasingly different from the one at the bond dissociation limit.

Measurement budget allocation and statistical errors

In this section we give a brief account of the effects of finite sample size on the accuracy of the expectation value estimates performed during the VQE and PCM-VQE procedure. The goal of this section is two-fold: on one hand we want to provide a practical recipe to estimate the number of shots N needed to achieve a desired accuracy on solvation quantities (and, in general, on any expectation value calculated from the VQE wavefunction); on the other hand we want to rationalize the magnitude of the errors at fixed number of shots for

different systems and solvation quantities.

The analysis we report explicitly refers to an error due to finite sampling for which it is exact to consider the variances of the expectation values. We point out that in presence of quantum computer noise it would be more appropriate to work explicitly with the mean squared error MSE(X) defined as

$$\mathrm{MSE}(X) = \mathrm{Var}(X) + \mathrm{Bias}(X)\,, \tag{S2}$$

where X is a random variable representing the expectation value, Var(X) is its variance and Bias(X) comprises the error due to the presence of the quantum computer noise.

We first consider the problem of estimating the number of optimal shots N needed to estimate the expectation value of an observable $\hat{O}$ within a desired accuracy ϵ. We follow Rubin *et al.*[11] who derived an optimal number of measurement for $\langle\hat{O}\rangle = \sum_\gamma h_\gamma P_\gamma$ given by

$$N = \frac{K}{\epsilon^2}\,, \tag{S3}$$

where the factor K is obtained taking into account the fluctuation of each independently measured Pauli word P_α needed to compute the desired expectation value:

$$K = \Big(\sum_B \sqrt{\sum_{\alpha,\beta\in B} h_\alpha h_\beta \mathrm{Covar}(P_\alpha, P_\beta)}\Big)^2 \tag{S4}$$

Here B is a label that runs over the different sets of non-commuting Pauli words and h_α, h_β are the corresponding matrix elements of the mapped operator $\hat{O}$.

We highlight that Eq. S4 is a direct consequence of error propagation theory and formalizes two important statements: (i) the number of shots increases as the number of independently measured Pauli words increases, (ii) the number of shots increases as the magnitude of the matrix elements of $\hat{O}$ increases.

In order to use Eq. S4 in practice we need to replace Covar(P_α, P_β) with a reasonable estimate. A common choice,[12,13] since $\mathrm{Var}(P_\alpha) = 1 - \langle P_\alpha\rangle^2$, is to set all the variances to 1

and all the covariances to zero. It is possible to show that this corresponds to assuming that the Pauli strings are sampled from a uniform distribution.[14]

With this assumption, K (and in turn the number of shots N) will depend only on the Frobenius norm of the operator $||O||$:

$$K \approx ||O||^2 = \sum_B \sum_{\alpha \in B} |h_\alpha|^2 \tag{S5}$$

Gonthier and coworkers[13] found numerically (for quantum chemistry ground state calculations on organic molecules at CCSD(T) level of accuracy) that putting covariances at zero overestimates K (and thus the required number of shots N) by a factor of 2, while assuming the variances to be all equal to 1 lead to a 20%-30% overestimation of K. Overall, their results show that using Eq. S3 together with the K estimate in Eq. S5 yields quite conservative (i.e., on the safe side) values of N.

To use what reported here in practice, if a given precision ϵ is sought, one has first to estimate the factor K summing all the squared $\hat{O}$ matrix elements corresponding to non-commuting Pauli strings and then to divide by ϵ^2 to obtain the corresponding number of samples N to collect.

Now we shall consider the error estimated on the total energy and total free energy. This is also relevant for the error on solvation free energies, which are obtained as differences of total free energy in solution and total energy in gas-phase. Suppose for example that an error of $\epsilon = 0.1$ Ha on the total energy (or free energy) for water at the STO-3G level is desired. The first step is to calculate $K = ||H||^2$ for water from eq. S5, that turns out to be $\approx 6 \cdot 10^3$. Then, eq. S3 gives a number of samples $N \approx 6 \cdot 10^5$.

The presented discussion can also be used to numerically explore how error estimates depend on the specific calculation, via the Hamiltonian norm and the number of non-commuting sets of Pauli words at fixed number of shots, see Tab. S2.

As we can see from such Table, the error increases both as the number of non commuting Pauli terms and the Hamiltonian norm increases. This is in accordance with Eq. S3 and Eq.

Table S2: Frobenius norm of the Hamiltonian $\hat{H}$, number of non-commuting sets of Pauli words B and statical errors ϵ for the molecules studied in this work at the STO-3G basis set level. Estimates computed according to Eq. S5. Values obtained for a number of samples $N = 8192$. All quantities are given in atomic units.

	$\|\|H\|\|$	B	ϵ
H_3^+	1.12	10	0.0124
BeH_2	17.7	17	0.195
H_2O	78.5	23	0.867

S5.

Turning now to the error on the polarization energy, that we identify in the main text as a robust solvation quantity also for noisy quantum computers, we note that the same procedure just described to estimate N to obtain a desired accuracy ϵ can be applied directly to the operator V_σ. Moreover, by using Eq. S4 we can also rationalize the different accuracy observed in estimating the solvation free energy and the polarization energy. Ultimately this should be ascribed to the fact that the number of independently measured Pauli strings is much smaller for the polarization energy alone than for the overall solvation free energy since in one case we deal with a one-electron operator while in the latter case we have to include all the measurements coming from the two-electrons part. More specifically, in Tab. S3 we show estimated sampling errors for $\frac{1}{2}V_\sigma$ (the operator needed to compute the polarization energy). The comparison of the magnitude of the errors reported in this Table with those in Tab. S2 confirms our qualitative analysis. Interestingly, the error for the smallest molecule H_3^+ here is the largest in the Table. This is not surprising, since H_3^+ is the only charged molecule in the set, and as a consequence the V_σ norm is the largest.

Table S3: Frobenius norm of the operator $\frac{1}{2}V_\sigma$, number of non-commuting set of Pauli words B and statistical errors ϵ for the molecules studied in this work at the STO-3G basis set level. Estimates computed according to Eq. S5. Values obtained for a number of samples $N = 8192$. All quantities are given in atomic units.

	$\frac{1}{2}\|\|V_\sigma\|\|$	B	ϵ
H_3^+	0.712	3	0.00786
BeH_2	0.0735	5	0.00081
H_2O	0.104	6	0.00115

Algorithmic complexity: PCM classical overhead

The proposed PCM-VQE algorithm is an hybrid one, combining a classical portion with a quantum one. In view of realistic simulations on future quantum computers, it is important to assess that the extension to PCM does not hamper potential quantum advantage from the VQE approach. The goal of this section is not to provide a thorough analysis of the algorithmic cost, which depends on the particular flavour of VQE considered, but to highlight the computational overhead due to the inclusion of solvation effects within the PCM framework.

Since we are considering an hybrid algorithm we estimate the overall cost $C = C_Q + C_C$ where C_Q is the cost associated with operations run on the quantum computer and C_C the classical cost to compute the molecular integrals .

Concerning C_Q, we note that gas-phase VQE requires evaluation of 1- and 2-RDM to obtain the expectation value of the molecular Hamitonian $\langle \hat{H}_0 \rangle$ (see Figure 1 in the main text). The 1-RDM is the only information from the quantum computer that is also needed to calculate $\langle \hat{V}_\sigma \rangle$, i.e., the additional part with respect to $\langle \hat{H}_0 \rangle$ to calculate the total free energy. Since the 1-RDM is calculated for the standard VQE as well, there is no quantum overhead for PCM-VQE with respect to gas-phase VQE (i.e., $C_Q^{\text{gas}} = C_Q^{\text{PCM}}$).

Now we move our analysis to the classical cost of the algorithm. Here the inclusion of solvation effects may potentially affect the algorithmic cost. Particularly, the standard molecular Hamiltonian is usually built computing the molecular one- and two-electron integrals.[15] On the other hand, inclusion of solvation effects calls for (i) computing the solvent response at each iteration $\mathbf{q}$ and (ii) calculating the solute-solvent interaction term $\langle \hat{V}_\sigma \rangle$ at each iteration. (i) Formally, computing the response charges implies the inversion of the PCM response matrix whose computational cost is $\mathcal{O}(N_{tess}^3)$. In practice it is possible to achieve a linear scaling with N_{tess} for this step adopting techniques such as the Fast Mul-

tipole Method (FMM) and parallelization.[16] In turn, N_{tess} is scaling with the size of the molecular surface, that is linear with the number of atoms N_{atoms} in the worst case of linear molecules. This is a negligible scaling w.r.t. the following contribution. (ii) Concerning the calculation of $\langle \hat{V}_\sigma \rangle$, at each step we have to contract the 1-RDM with the $\mathbf{v}_{rs}$ array a step costing $\mathcal{O}(N_a^2 N_{tess}) = \mathcal{O}(N_a^2 N_{\text{atoms}})$ additional operations. Reasonably assuming that N_a is scaling linearly with N_{atoms}, this step has a $\mathcal{O}(N_a^3)$ scaling. This is still a better scaling than the cost of the contraction of the 2-RDM with the gas-phase bielectronic integrals (that dominates the gas-phase C_C), where a sum over four index is needed ($\mathcal{O}(N_a^4)$). As such the scaling of C_C^{PCM} will be at most equal to that of C_C^{gas}. Of course, any strategy to improve such $\mathcal{O}(\text{N}_a^4)$ scaling (e.g., use of molecular symmetry, prescreening of the molecular integrals) can also be used to improve the scaling of the classical PCM term.

In conclusion, the overhead of the PCM extension to VQE is null for the quantum part, and does not worsen the scaling of the classical part with respect to gas-phase calculations; a potential quantum advantage of gas-phase VQE is therefore unhampered by the inclusion of the PCM solvent. The bottleneck remains to be the solution of the electronic structure problem.

Quantum circuits

(a)

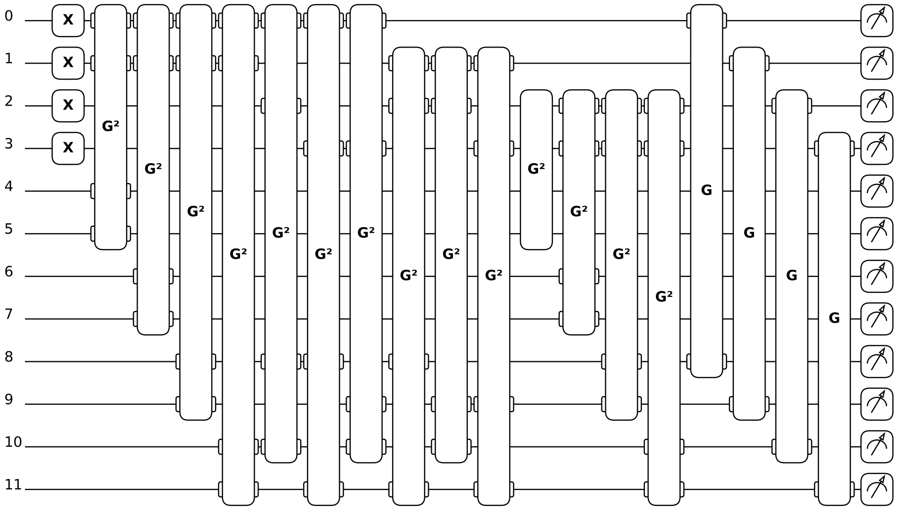

(b)

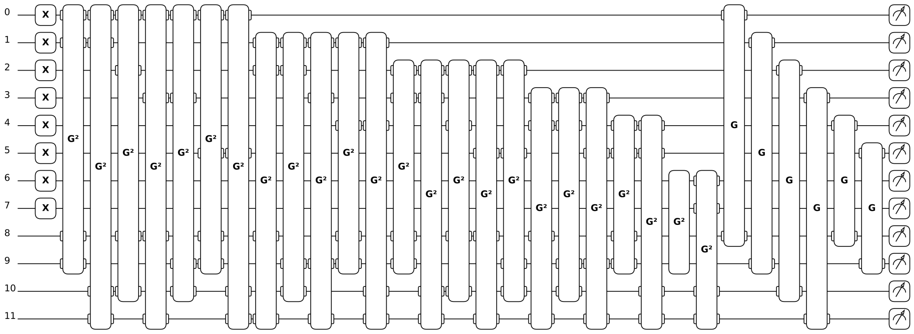

Figure S5: Quantum circuits used for the PCM-VQE simulations for the BeH_2 (a) and H_2O (b) molecules. Please note the difference w.r.t. the circuit reported in the main text for H_3^+. The space of multiple excitations is realised by applying more Givens rotations (18 and 30 for BeH_2 and H_2O respectively). We also note the presence of single excitation operators missing in the H_3^+ circuit as a result of the adaptive procedure that discards irrelevant excitations. Labels G and G^2 refer to single- and double-excitation unitaries implemented as rotations in the subspace spanned by two or four qubits. Figure obtained using the quantum circuit drawer function as implemented in PennyLane.[17]

References

(1) https://github.com/davidecast/PCM VQE, Accessed by date:10/11/2022.

(2) Babbush, R.; McClean, J.; Wecker, D.; Aspuru-Guzik, A.; Wiebe, N. Chemical basis of Trotter-Suzuki errors in quantum chemistry simulation. *Physical Review A* **2015**, *91*,

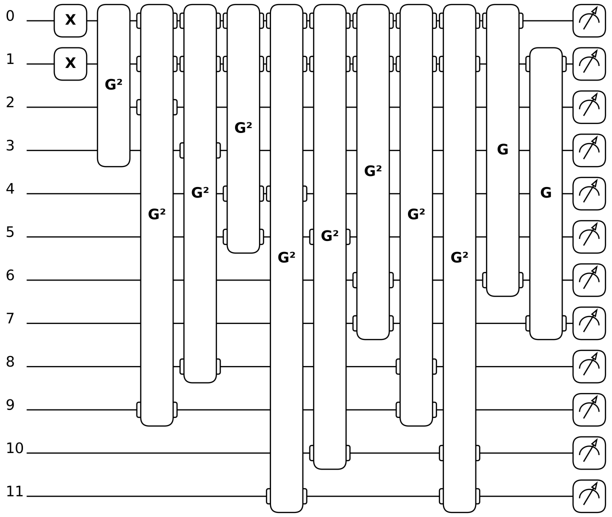

Figure S6: Quantum circuit used for the PCM-VQE simulation of H_3^* with a 6-31G basis set. Compared to the circuit shown in the main text (referring to a STO-3G calculation) the number of qubits increases from 4 to 12. The number of Givens excitations included in the adaptive ansatz is 11.

022311.

(3) Peruzzo, A.; McClean, J.; Shadbolt, P.; Yung, M.-H.; Zhou, X.-Q.; Love, P. J.; Aspuru-Guzik, A.; O'brien, J. L. A variational eigenvalue solver on a photonic quantum processor. *Nature communications* **2014**, *5*, 1–7.

(4) Shen, Y.; Zhang, X.; Zhang, S.; Zhang, J.-N.; Yung, M.-H.; Kim, K. Quantum implementation of the unitary coupled cluster for simulating molecular electronic structure. *Physical Review A* **2017**, *95*, 020501.

(5) Cammi, R. Quantum cluster theory for the polarizable continuum model. I. The CCSD level with analytical first and second derivatives. *The Journal of chemical physics* **2009**, *131*, 164104.

(6) Smith, D. G.; Burns, L. A.; Simmonett, A. C.; Parrish, R. M.; Schieber, M. C.; Galvelis, R.; Kraus, P.; Kruse, H.; Di Remigio, R.; Alenaizan, A., et al. PSI4 1.4:

Open-source software for high-throughput quantum chemistry. *The Journal of chemical physics* **2020**, *152*, 184108.

(7) Sim, S.; Johnson, P. D.; Aspuru-Guzik, A. Expressibility and entangling capability of parameterized quantum circuits for hybrid quantum-classical algorithms. *Advanced Quantum Technologies* **2019**, *2*, 1900070.

(8) Choquette, A.; Di Paolo, A.; Barkoutsos, P. K.; Sénéchal, D.; Tavernelli, I.; Blais, A. Quantum-optimal-control-inspired ansatz for variational quantum algorithms. *Physical Review Research* **2021**, *3*, 023092.

(9) Holmes, Z.; Sharma, K.; Cerezo, M.; Coles, P. J. Connecting ansatz expressibility to gradient magnitudes and barren plateaus. *PRX Quantum* **2022**, *3*, 010313.

(10) Delgado, A.; Arrazola, J. M.; Jahangiri, S.; Niu, Z.; Izaac, J.; Roberts, C.; Killoran, N. Variational quantum algorithm for molecular geometry optimization. *Phys. Rev. A* **2021**, *104*, 052402.

(11) Rubin, N. C.; Babbush, R.; McClean, J. Application of fermionic marginal constraints to hybrid quantum algorithms. *New Journal of Physics* **2018**, *20*, 053020.

(12) Crawford, O.; van Straaten, B.; Wang, D.; Parks, T.; Campbell, E.; Brierley, S. Efficient quantum measurement of Pauli operators in the presence of finite sampling error. *Quantum* **2021**, *5*, 385.

(13) Gonthier, J. F.; Radin, M. D.; Buda, C.; Doskocil, E. J.; Abuan, C. M.; Romero, J. Measurements as a roadblock to near-term practical quantum advantage in chemistry: Resource analysis. *Physical Review Research* **2022**, *4*, 033154.

(14) Gokhale, P.; Angiuli, O.; Ding, Y.; Gui, K.; Tomesh, T.; Suchara, M.; Martonosi, M.; Chong, F. T. Minimizing state preparations in variational quantum eigensolver by partitioning into commuting families. *arXiv preprint arXiv:1907.13623* **2019**,

(15) Helgaker, T.; Jorgensen, P.; Olsen, J. *Molecular electronic-structure theory*; John Wiley & Sons, 2014.

(16) Scalmani, G.; Barone, V.; Kudin, K. N.; Pomelli, C. S.; Scuseria, G. E.; Frisch, M. J. Achieving linear-scaling computational cost for the polarizable continuum model of solvation. *Theoretical Chemistry Accounts* **2004**, *111*, 90–100.

(17) Bergholm, V.; Izaac, J.; Schuld, M.; Gogolin, C.; Alam, M. S.; Ahmed, S.; Arrazola, J. M.; Blank, C.; Delgado, A.; Jahangiri, S., et al. Pennylane: Automatic differentiation of hybrid quantum-classical computations. *arXiv preprint arXiv:1811.04968* **2018**,

TOC Graphic

Some journals require a graphical entry for the Table of Contents. This should be laid out "print ready" so that the sizing of the text is correct.
Inside the `tocentry` environment, the font used is Helvetica 8 pt, as required by *Journal of the American Chemical Society*.
The surrounding frame is 9 cm by 3.5 cm, which is the maximum permitted for *Journal of the American Chemical Society* graphical table of content entries. The box will not resize if the content is too big: instead it will overflow the edge of the box.
This box and the associated title will always be printed on a separate page at the end of the document.
